# Battling brain-eating amoeba: Enigmas surrounding immunity to *Naegleria fowleri*

**DOI:** 10.1371/journal.ppat.1008406

**Published:** 2020-04-23

**Authors:** E. Ashley Moseman

**Affiliations:** Department of Immunology, Duke University School of Medicine, Durham, North Carolina, United States of America; Children's Hospital of Philadelphia, UNITED STATES

## Introduction

Free-living amoebas (FLA) are remarkable single-cell engines, foraging their way through a range of environments. However, occasionally these amoebas find themselves within a human host, and an unusual and deadly opportunistic infection unfolds. The FLA *Naegleri fowleri* is the causative agent for an invasive and lethal form of meningoencephalitis known as primary amoebic meningoencephalitis (PAM). According to the CDC, 147 patients have been diagnosed with PAM in the United States since 1962. Yet, because distinguishing PAM from other types of meningitis and encephalitis can be difficult, it’s likely that many cases of PAM are simply never identified, especially in areas of the world with under-resourced healthcare systems. Because *N*. *fowleri* is a thermophilic organism, rising global temperatures will prolong growth seasons and expand compatible habitats. In fact, although *N*. *fowleri* infection and PAM are not a nationally notifiable disease, there is evidence that the latitude of reported infections has broadened over the past 10 to 15 years [1]. When paired with potentially increased water recreation, a warming climate may facilitate a collision course of amoebic growth and human activity.

## Collision course: *N*. *fowleri* encounter with mammalian hosts

As a free-living amoeba, *N*. *fowleri* is fully capable of reproducing without a host, and mammals are certainly not a requisite step in the *N*. *fowleri* life cycle. Indeed, *N*. *fowleri* are found in warm fresh water across the globe, making human contact commonplace and typically benign. *N*. *fowleri* can exist in 3 forms: a dormant cyst form, a migratory flagellate, and the pathogenic trophozoite that feeds and divides. PAM occurs when trophozoites access the nasal turbinates and cross the olfactory epithelium (OE) to enter olfactory nerve bundles and migrate into the brain, where they provoke an intense inflammatory reaction and lethal increases in intracranial pressure. Even though *N*. *fowleri* infection is purely opportunistic, unlike many other opportunistic infections, it is not associated with immunocompromised individuals; on the contrary, PAM patients are typically young and seemingly healthy at the time of exposure [2]. The sudden infection and death of otherwise healthy young people underlies the 2 biggest mysteries surrounding *N*. *fowleri* infections: Why are some people infected, while others are not, when exposed to seemingly similar conditions? Why is nasal *N*. *fowleri* exposure the only route with dire consequences? These 2 unknowns are probably linked because although there are animal models of visceral/peripheral naegleriosis [3], human peripheral infection is virtually unknown, even when people undoubtedly swallow parasites or have exposed open wounds.

**Fig 1:**
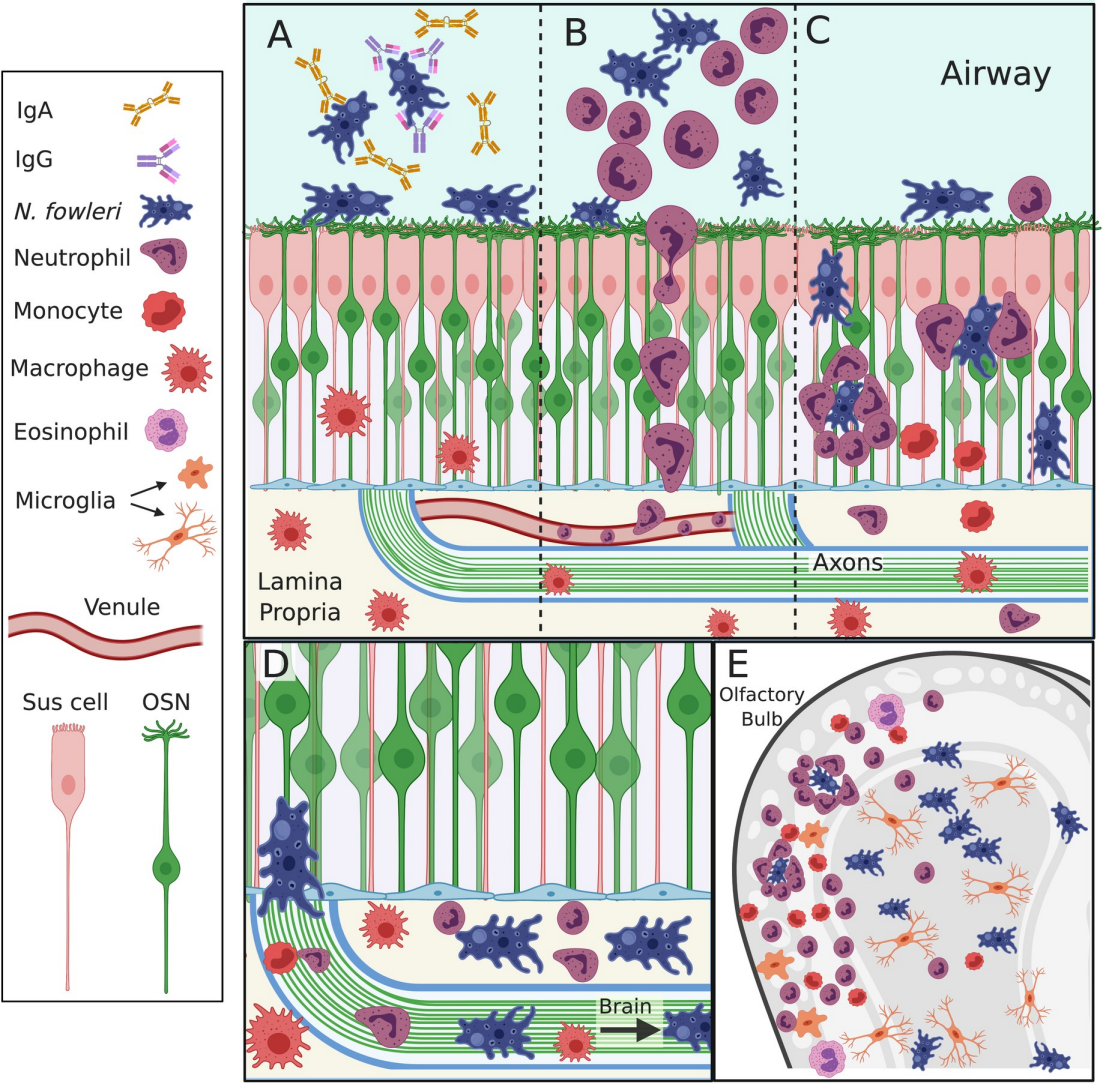
Model of early olfactory immune response to *N*. *fowleri* infection. (**A)** Water entry into the olfactory turbinates delivers *N*. *fowleri* to the olfactory epithelial surface. IgA, IgM, and IgG secreted within the airway potentially interfere with attachment to the epithelial surface, and the parasite is washed harmlessly away. **(B)** If this antibody response is insufficient, *N*. *fowleri* interaction with the olfactory surface results in an early (approximately 10 hours) and robust innate response. This early cellular response is characterized by inflammatory cell entry, particularly neutrophils, into the airway space, where they reduce the number of amoebas through effector mechanisms or mechanically inhibiting amoeba interaction with the epithelial surface. **(C)** When initial mechanisms fail to prevent parasite olfactory invasion, innate responses within the OE can be triggered. These responses involve macrophage and monocyte lineage cells but are again dominated by neutrophils. However, some parasites evade immune detection at this point, allowing them to continue invading deeper into the lamina propria. *N*. *fowleri* invasion of the lamina propria results in the parasite gaining entry into the olfactory nerve bundles. **(D)** These structures serve as conduits for OSNs axons to reach the brain, yet they now become a direct parasite pathway into the brain. Occasionally, innate cells will recognize amoeba within the axon bundles, but numerous amoebas make their way into the brain. **(E)** Once in the brain, *N*. *fowleri* proliferate and eventually provoke a massive inflammatory infiltration consisting of neutrophils, monocytes, and eosinophils that drives lethality. IgA, immunoglobulin A; IgG, immunoglobulin G; IgM, immunoglobulin M; IgM; *N*. *fowleri*, *Naegleria fowleri*; OE, olfactory epithelium; OSN, olfactory sensory neurons.

Therefore, a critical component of *N*. *fowleri*’s lethal opportunism likely lies in the barrier being breached within nasal turbinates. There is evidence that *N*. *fowleri* can penetrate the respiratory epithelium [4], but our experiments suggest this is rather uncommon. However, penetration of adjacent OE provides *N*. *fowleri* with immediate access to olfactory sensory axon bundles [5] that serve as de facto “tunnels” for amoebas to migrate directly into the brain (Fig 1D), bypassing conventional central nervous system (CNS) barrier protections. Although it has been suggested that amoeba actively chemotax toward brain tissue [6], it may be that the anatomical structures of the OE simply provide a path of least resistance that lead to the brain. In any event, anatomy alone cannot explain why immune mechanisms sufficient elsewhere in the periphery fail within the OE. Might differences in the immune response partially explain why certain individuals develop PAM? Studies of human serum and mucosal antibody titers have found widespread evidence of anti-Naegleria immune responses resulting from subclinical *N*. *fowleri* exposure [7–10]. These immune responses may arise after a nonolfactory exposure or olfactory clearance of less pathogenic strains of *N*. *fowleri*. Although there is no evidence that overt immunodeficiency predisposes toward *N*. *fowleri* infection, the presence of detectable but variable immune responses suggests that differences in innate and adaptive immunity contribute to developing PAM.

## Hurdles to understanding immunity to Naegleria

Because there remain no effective clinical treatments for PAM, defining the mechanisms underlying the early immune failure and the factors that precipitate the subsequent fulminant inflammation may suggest improvements in clinical care. Deciphering these immune mechanisms and retrospectively understanding the human immune response is particularly challenging because of the swiftness of PAM and the rarity of surviving patients. Luckily, animal models of PAM appear remarkably similar to human infections and offer a powerful tool for characterizing how the immune system perceives and responds to *N*. *fowleri*. While in vitro experiments have revealed many pathogenic mechanisms employed by *N*. *fowleri* [11], a shortage of mechanistic in vivo studies on the immune response to *N*. *fowleri* has left many basic questions unanswered. Does breach of the olfactory barrier unequivocally result in death, or must there also be a combined failure of adaptive and innate mechanisms to result in PAM? What protective immune responses could prevent individuals from being infected in the first place? Which cells and mechanisms are critical for killing *N*. *fowleri* in vivo? Is the immune response beneficial to the host at all or simply causing further damage? We cannot fully answer these questions, but this review highlights our current understanding as well as what remains unclear.

### Innate immune response to Naegleria: Intense and incomplete

Immune cell infiltration into the CNS is closely associated with the edema that drives herniation and death in PAM patients. However, depletion of CD11b-expressing cells hastened death in animal models, suggesting that neutrophils and infiltrating hematopoietic cells provide an important source of antiamoebic pressure [12], even if overexuberance may contribute to lethality. In contrast to the intense inflammation of end-stage PAM, the initial invasive process of the amoeba is remarkably uninflammatory. Rojas-Hernández and colleagues characterized a very early cellular exudate within the nasal turbinates hours after infection [4], yet parasites then invaded the OE and followed the olfactory axon tracts toward the brain without eliciting significant numbers of innate inflammatory cells [5,13] (Fig 1B–1D). Amoeba are eventually detected, and the subsequent infiltration of neutrophils, eosinophils, monocytes, and macrophages [13] ignites a cascade of hemorrhage and lytic necrosis within the brain 3 to 4 days after infection. Although inflammation ultimately characterizes this disease, the early failure to detect parasites and employ effective antiamoebic mechanisms is particularly noteworthy. How phagocytic parasites could migrate undetected within the CNS is difficult to reconcile with conventional viewpoints on innate cell recruitment to sites of cellular injury. This capacity of individual parasites to enter the CNS without immune detection likely plays a crucial role in the failure to control amoeba and prevent lethality.

How then does the immune system recognize amoebic invasion? Unlike bacterial or viral pathogens, *N*. *fowleri* is eukaryotic, and most mammalian pattern recognition receptors will not recognize it as foreign. Complement activation, particularly that mediated by antibody, can drive enhanced neutrophil activity against the amoeba [14,15]. Additionally, complement cleavage products are known to serve as a chemotactic impetus for immune cell recruitment. However, pathogenic variants of *N*. *fowleri* are resistant to downstream complement mediated lysis [16], and there is scant in vivo evidence that complement is critical to *N*. *fowleri* containment. Because neutrophils show no intrinsic chemotactic response toward *N*. *fowleri* [15], how is the alarm bell rung? Complement activation may contribute, but beyond that, it’s unclear which cell types can recognize the presence of the amoeba within the airway, OE, or brain. Amoebas likely damage host cells as they break down intercellular matrices or tear off host cell membranes to feed via trogocytosis [17]. This “bull in a china shop” approach should result in extracellular ATP release from host cells, yet there are few data to support purinergic receptor based recruitment [18] of innate cells to *N*. *fowleri* within the OE or CNS. Several studies have indicated that antiamoebic neutrophil responses are regulated by cytokines produced by other cells [14,19].Tumor necrosis factor alpha (TNFα), in particular, has been shown to “license” or “awaken” neutrophils to engage and target amoebas for destruction, likely through mechanisms that are dependent upon myeloperoxidase, superoxide formation, or neutrophil extracellular trap (NET) release [19–23]. Individually, immune cells stand little chance against *N*. *fowleri*; however, neutrophils can employ a group attack technique in which numerous neutrophils encircle an individual amoeba to centrally target effector activity [13,14] (Fig 1C and 1E). How neutrophils are able to locate and target the amoeba is unclear, but the failure of sentinel cells to detect *N*. *fowleri* and elicit a licensing stimulus such as TNFα may be partially responsible for *N*. *fowleri*’s ultimate immune evasion. Indeed, bypassing sentinel recognition through injection of a potent TNFα inducer (muramyl dipeptide) protects animals from PAM even after disease manifestations have begun [24]—a truly remarkable finding that suggests it may be possible to augment or attenuate specific immune functions to tailor a less pathologic and more protective immune response [25].

### Adaptive immunity to Naegleria—A critical mediator of prevention?

Studying the adaptive immune response to *N*. *fowleri* infection in PAM patients is especially difficult because of the rapidly lethal disease course. In vitro studies have shown that *N*. *fowleri* can rapidly internalize surface binding antibodies [26,27], a behavior that has been suggested to undermine the utility of the humoral response. Nonetheless, in vivo, amoeba may initially internalize antibodies, but serum will continuously replenish local antibody concentrations and drive effector activity. Several immunization strategies utilizing amoebic lysate, cell culture supernatant, live amoeba, fixed amoeba, and specific protein via different inoculation routes have resulted in measurable antibody titers and varying degrees of protection [2]. Immune serum transfer experiments have confirmed that circulating antibodies are the dominant protective adaptive immune mechanism [28,29] (Fig 1A). In addition, intrathecal therapeutic monoclonal antibody administration has been shown to prolong survival in animals [30]. Antibodies potentially impinge on amoebic lifestyle in several ways depending upon their isotype: they can opsonize to facilitate fragment crystallizable (Fc) receptor–mediated phagocytosis or effector activity, activate complement to target immune cells to the amoeba, as well as promote direct lysis. However, analysis of human sera has found the majority of anti-Naegleria antibodies are directed toward internal structures [31], rather than protective surface antigens. And of those, the primary antibody isotype generated upon *N*. *fowleri* infection in humans is immunoglobulin M (IgM) [2]. Although IgM can drive *N*. *fowleri* agglutination [32] and complement activation, IgM’s high molecular weight (approximately 900 kD) may impede crossing the blood–brain barrier to access the infected brain.

An IgM bias in the *N*. *fowleri* humoral response could be the result of several factors. Amoebic surface antigens may favor T-independent responses (as is this case with bacterial polysaccharides [33]), or there may be defects in the Naegleria-specific CD4+ repertoire, priming, and functions that facilitate antibody class switch. Cell-mediated immunity against *N*. *fowleri* has been observed in the form of delayed-type hypersensitivity [34], but there has not been a careful dissection of amoeba-specific CD4+ T-cell functionality after infection or vaccination. In immunized animals, IL-4 levels correlated with survival—an effect dependent upon STAT6, suggesting a role for Th2 cells in facilitating an antiamoebic vaccine response [35]. Tissue-specific exposure is known to guide immune bias, and indeed, intranasal vaccinations have yielded substantial increases in *N*. *fowleri*-binding IgG and IgA antibody titers, which correlated with protection from lethal challenge [35,36] (Fig 1A). And yet, we fundamentally don’t understand how *N*. *fowleri*–specific adaptive immune responses are generated, specifically, how and where antigen-presenting cells acquire amoebic antigen, as well as how these cells could coordinate an adaptive immune response within the relevant tissues.

There are many open questions surrounding fundamental immunological processes during *N*. *fowleri* infection (Table 1). The protective potential of antibody responses is clear; however, dissection of the relevant antibody isotypes and Fc receptors that provide protective immunity is still needed to guide vaccine design or immunotherapeutic approaches. And while antibody titers are easy to measure, addressing the roles for other lymphocytes such as natural killer (NK) cells, NKT cells, gamma/delta T cells, or even potentially CD8+ T cells requires comprehensive mechanistic in vivo studies. This will require reversing a historical lack of funding for basic research into the host response to *N*. *fowleri* and other free-living amoebic pathogens but will catalyze transformative changes in the prophylactic and therapeutic clinical options for a devastating disease.

**Table 1:** 

Key Observations	Selected References	Open Questions
Innate immune cells provide resistance to amoeba.	[12,22,24]	What roles do specific cell types play in recognition and effector function; effector mechanisms?
Antibodies against *N*. *fowleri* can be protective.	[28–30]	Which classes and functions of antibody are critical at early and late stages of infection?
Healthy humans commonly have *N*. *fowleri* reactive antibodies.	[7–9,26]	Do human antibodies bind conserved epitopes across *N*. *fowleri* strains? Are conserved antigens (surface or intracellular) targeted? What is the antibody isotype bias between individuals?

N. fowleri, Naegleria fowleri

## Concluding remarks

It is impossible to know how frequent “subclinical” *Naegleria* infection is, though there is evidence that individuals from warmer southern states with more presumed *Naegleria* exposure have more serum antibody activity [32]. It’s possible that asymptomatic *Naegleria* infections occur with regularity but are simply contained and cleared [37]. However, this is little solace for those few individuals at the confluence of exposure, insufficient immunity, and luck. Understanding the role of immunity in preventing PAM should shed light on a frustratingly fundamental question asked by the families of PAM victims: Why?
